# Microglial exosome miR‐124‐3p in hippocampus alleviates cognitive impairment induced by postoperative pain in elderly mice

**DOI:** 10.1111/jcmm.18090

**Published:** 2023-12-23

**Authors:** Erliang Kong, Xuqiang Geng, Feixiang Wu, Wei Yue, Yuming Sun, Xudong Feng

**Affiliations:** ^1^ Department of Anesthesiology The 988th Hospital of Joint Logistic Support Force of Chinese People's Liberation Army Zhengzhou China; ^2^ Department of Rheumatology and Immunology, Changzheng Hospital Second Affiliated Hospital of Naval Medical University Shanghai China; ^3^ Department of Intensive Care Unit, Shanghai Eastern Hepatobiliary Surgery Hospital Third Affiliated Hospital of Naval Medical University Shanghai China; ^4^ Department of Anesthesiology, Shanghai Eastern Hepatobiliary Surgery Hospital Third Affiliated Hospital of Naval Medical University Shanghai China

**Keywords:** cognitive impairment, elderly, exosome, hippocampus, microglia, miR‐124‐3p, postoperative pain

## Abstract

Cognitive impairment induced by postoperative pain severely deteriorates the rehabilitation outcomes in elderly patients. The present study focused on the relationship between microglial exosome miR‐124‐3p in hippocampus and cognitive impairment induced by postoperative pain. Cognitive impairment model induced by postoperative pain was constructed by intramedullary nail fixation after tibial fracture. Morphine intraperitoneally was carried out for postoperative analgesia. Morris water maze tests were carried out to evaluate the cognitive impairment, while mRNA levels of neurotrophic factors (BDNF, NG) and neurodegenerative biomarker (VILIP‐1) in hippocampus were tested by *q*‐PCR. Transmission electron microscope was used to observe the axon degeneration in hippocampus. The levels of pro‐inflammatory factors (TNF‐α, IL‐1β, IL‐6), the levels of anti‐inflammatory factors (Ym, Arg‐1, IL‐10) and microglia proliferation marker cyclin D1 in hippocampus were measured to evaluate microglia polarization. Bioinformatics analysis was conducted to identify key exosomes while BV‐2 microglia overexpressing exosome miR‐124‐3p was constructed to observe microglia polarization in vitro experiments. Exogenous miR‐124‐3p‐loaded exosomes were injected into hippocampus in vivo. Postoperative pain induced by intramedullary fixation after tibial fracture was confirmed by decreased mechanical and thermal pain thresholds. Postoperative pain induced cognitive impairment, promoted axon demyelination, decreased BDNF, NG and increased VILIP‐1 expressions in hippocampus. Postoperative pain also increased pro‐inflammatory factors, cyclin D1 and decreased anti‐inflammatory factors in hippocampus. However, these changes were all reversed by morphine analgesia. Bioinformatics analysis identified the critical role of exosome miR‐124‐3p in cognitive impairment, which was confirmed to be down‐regulated in hippocampus of postoperative pain mice. BV‐2 microglia overexpressing exosome miR‐124‐3p showed decreased pro‐inflammatory factors, cyclin D1 and increased anti‐inflammatory factors. In vivo, stereotactic injection of exogenous miR‐124‐3p into hippocampus decreased pro‐inflammatory factors, cyclin D1 and increased anti‐inflammatory factors. The cognitive impairment, axon demyelination, decreased BDNF, NG and increased VILIP‐1 expressions in hippocampus were all alleviated by exogenous exosome miR‐124‐3p. Microglial exosome miR‐124‐3p in hippocampus alleviates cognitive impairment induced by postoperative pain through microglia polarization in elderly mice.

## 1 INTRODUCTION

Elderly patients usually are sensitive to surgical trauma, stress, anaesthesia or pain because of their irreversible degeneration in central nervous system (CNS), which greatly increases the incidence of postoperative cognitive impairment, especially after receiving cardiovascular, orthopaedic or cerebrovascular surgeries.^1^ Postoperative cognitive impairment is characterized by the disorders in consciousness, cognition, orientation, thinking, memory or sleep in patients after anaesthesia and surgery, alongside social impairment.^2^ Patients with postoperative cognitive impairment often exhibit anxiety, insanity, loss of learning and memory ability. The incidence of postoperative cognitive impairment in elderly patients was 25.8% in a week and 9.9% 3 months after noncardiac surgery, while the incidence was ranging from 40% to 70% in a week and 14%–30% 3 months after cardiac surgery.^3^, ^4^ In addition, approximately 30% to 60% of elderly patients exhibit delirium or cognitive dysfunction in a week after cardiovascular surgery.^5^, ^6^

Postoperative pain is a series of complex pathophysiological reactions caused by surgical trauma and significantly impairs the recovery of organ functions. The mechanisms involved in postoperative pain are inflammation, nerve damage and synaptic remodelling. In the cognitive process, hippocampus plays a critical role in regulating emotion, learning and memory formation.^7^ Postoperative pain induced by surgical trauma significantly causes stress and inflammatory responses, and induces the release of inflammatory cytokines in hippocampus.^8^ The intricate interplay between the peripheral immune system and the CNS plays a pivotal role in this phenomenon.^9^ Surgical trauma induces the release of pro‐inflammatory cytokines and chemokines at the site of tissue injury, initiating a local inflammatory response. These inflammatory mediators can activate peripheral immune cells, such as macrophages and monocytes, which then infiltrate the CNS and further contribute to the neuroinflammatory cascade.^10^, ^11^ In the hippocampus, microglia, the resident immune cells of the brain, play a key role in sensing and responding to these inflammatory signals.^12^ Microglia activation is a complex process involving the transformation of microglia into an activated phenotype, characterized by morphological changes and the release of pro‐inflammatory cytokines, such as tumour necrosis factor α (TNF‐α) and interleukin‐1β (IL‐1β). This transition is regulated by various molecular mechanisms, including the activation of toll‐like receptors (TLRs) and nucleotide‐binding oligomerization domain‐like receptors (NLRs), as well as the downstream nuclear factor‐κB (NF‐κB) and mitogen‐activated protein kinase (MAPK) signalling pathways.^13^, ^14^

Growing evidence suggests that postoperative pain may play a crucial role in the development of cognitive impairment after surgery. Several studies have demonstrated a close association between postoperative pain intensity and the severity of cognitive decline.^15^, ^16^ Pain‐induced neuroinflammation has been implicated as a key contributor to cognitive impairment.^17^ In response to surgical trauma and tissue injury, microglial activation occurs, leading to the release of pro‐inflammatory cytokines and chemokines, which can trigger a cascade of neuroinflammatory responses.^18^ Microglia, which is particularly sensitive to pathological damage, can respond immediately to infection, inflammation and nerve damage. Under normal conditions, microglia are in the resting M0 state with strong plasticity, they can polarize to M1 state when face stress. Bacteria, viruses, macrophages, toll‐like receptors and CD80 can all promote the M1 polarization of microglia, inducing the release of TNF‐α, IL‐1β, IL‐6, inducible nitric oxide synthase, prostaglandin E2 and other pro‐inflammatory mediators.^19^, ^20^ These changes subsequently change the structures and functions of neuronal synapses and microenvironments.^21^, ^22^ Nevertheless, M2 polarization of microglia is beneficial to the secretion of IL‐4, IL‐10, transforming growth factor‐β (TGF‐β) and other anti‐inflammatory factors, which is crucial for neural restoration.^23^ Brain‐derived neurotrophic factor (BDNF), neurogranin (NG) and neurodegenerative biomarker visinin‐like protein‐1 (VILIP‐1) are also greatly affected by microglia polarization.^24^ The transition from M1 to M2 phenotype can be influenced by various factors, including the microenvironment and signalling molecules.^25^ Several studies have suggested that certain cytokines, such as IL‐4 and IL‐13, can induce M2 polarization in microglia.^26^, ^27^ Additionally, other factors like TGF‐β, glucocorticoids and extracellular vesicles, such as exosomes, have been implicated in promoting M2 phenotype transformation. These molecules can activate specific signalling pathways, such as STAT6 and SMAD3, leading to the upregulation of M2 markers and the downregulation of M1‐associated markers.^28^, ^29^ Therefore, microglia polarization around hippocampal neurons can easily affect cognitive impairment in elderly patients.

Nowadays, plentiful researches focused on the regulatory functions of exosomes in microenvironments around neurons.^30^ Containing plentiful specific proteins, lipids and genetic material, exosomes participate in signal transmission, internal environmental stability, immune response, neurodegeneration and cell death, which are closely related to pathological processes.^31^ Our preliminary experiments identified a highly specific exosome miRNA miR‐124‐3p in the CNS, which was widely involved in microglia regulation, inflammatory responses, neuronal development and regeneration. The related downstream genes of miR‐124‐3p participate in autophagy, inflammation and apoptosis, indicating the close relationship between miR‐124‐3p and neural activities.^32^ Previous studies have confirmed that microglia in hippocampus can secrete various types of exosomes into microenvironments to regulate microglia polarization and neural degeneration, which is essential in cognitive impairments.^33^ The present study focused on the relationship between microglial exosome miR‐124‐3p in hippocampus and cognitive impairment induced by postoperative pain in elderly mice, aiming at exploring the potential protective mechanisms of exosome miR‐124‐3p in cognitive impairment, and advancing the understanding of enhanced recovery after surgery in elderly patients.

## 2 MATERIALS AND METHODS

### 2.1 Animals and drugs

Wild‐type C57BL/6J elderly male mice (18 months) were provided by the Experimental Animal Center of Naval Medical University (Shanghai, China). All mice were housed in a pathogen‐free environment (24°C room temperature and 50% humidity) under a 12/12‐h light/dark cycle, with food and water ad libitum. Maximal efforts were made to minimize the number of animals used in the study and reduce animal suffering. The study also acquired the permission from the animal ethics committee of Naval Medical University. Pentobarbital sodium was dissolved in normal saline and administrated intraperitoneally at 50 mg/kg for anaesthesia before surgery. For postoperative analgesia, morphine (MOR) was dissolved in normal saline and administrated intraperitoneally at 0.1 mg/kg at 11:00 in the morning on Days 0–2 for the first three consecutive days after surgery.

### 2.2 Surgery of postoperative pain model

Postoperative pain model (M) was established by intramedullary nail fixation after tibial fracture at 10:00 in the morning on Day 0. Mice were divided into three groups: control (Naïve + Veh), postoperative pain model with vehicle for postoperative analgesia (M + Veh), postoperative pain model with morphine for postoperative analgesia (M + MOR). After anaesthesia with pentobarbital sodium intraperitoneally, mice were fixed on a heating pad at a surgical temperature of 37°C. The skin was incised, and muscles were separated below the knee to expose the tibia; then, artificial mid‐shaft tibial fracture was induced; and a 0.2 mm nail was inserted into the medullary cavity to fix the fractured ends intramedullarily. Finally, the incision was sutured in multiple layers and 80 thousand units of penicillin were administrated intramuscularly to avoid infection. Mice were allowed to recover on the heating pad for 1 h and receive postoperative analgesia with vehicle or morphine (MOR) at 11:00 in the morning on Days 0–2 for the first three consecutive days after surgery according to subject design.

### 2.3 Measurements of mechanical and thermal pain thresholds

Mechanical and thermal pain thresholds were assessed at 8:00 in the morning on Days 0, 3, 7 and 14 after surgery in a quiet environment between 09:00 and 11:00. Prior to testing, mice were given a 2‐h acclimatization period in Plexiglas™ cages with wire mesh flooring. Von Frey filaments (North Coast Medical, Gilroy, CA, USA) of 0.07 and 0.4 g were gently applied to the plantar surface of the hind paw, targeting the area innervated by the sciatic nerve. The 0.07 g and 0.4 g Von Frey filaments were utilized to assess pain allodynia and hyperalgesia, respectively. Each mouse underwent 10 trials with a 10‐min interval between each trial. A positive response was recorded when a mouse exhibited a rapid withdrawal response. The total number of positive responses was tallied, and the percentage was calculated as the mechanical withdrawal frequency. Subsequently, the mice were transferred to test cages with a transparent glass plate for thermal pain threshold measurement. After a period of acclimatization, a heating device was applied under the hind paw with a fixed heat intensity and a 30‐s cut‐off time to prevent tissue damage. A positive response was recorded when a mouse displayed a brisk withdrawal, and the duration of withdrawal was considered as the withdrawal latency. The withdrawal latency of the hind paw was assessed three times for each mouse with a 5‐min interval between assessments, and the average value was taken as the thermal threshold.

### 2.4 Morris Water Maze test

Morris water maze (XR‐XM101, Shanghai) was used to evaluate the cognitive impairment at 9:00 in the morning on Days 0, 3, 7 and 14 after surgery induced by postoperative pain in elderly mice. According to the pharmacokinetics of morphine, by the third day of postoperative testing, the drug has been completely metabolized in the mice, and as a result, it does not influence the outcomes of the Morris water maze tests. The tests were conducted in a large circular pool filled with water to a depth of 35 cm, and the temperature was maintained at 25°C. A platform was submerged 1 cm below the surface of the water and placed in the middle of the same quadrant throughout the training phase. In the training phase, mice were allowed to swim freely for 90 s to search the submerged platform. A 30‐s rest on the platform was allowed for mice if they found the platform successfully in 90 s; otherwise, the mice were guided to the platform for rest. After trained for six consecutive days before surgery, the learning process consisted of three consecutive trials with 5‐min intervals and the time to find the platform was recorded as the escape latency to assess learning ability. In the space exploration phase, the platform was removed from the pool and mice were put into water. The swimming route was recorded, the time spent in the platform quadrant and the frequency that mice passed the position of platform were noted as spatial memory function. These parameters are crucial indicators of cognitive function, as they reflect the ability of mice to learn and remember spatial information. Mice with cognitive impairment showed increased escape latency and decreased duration in target quadrant and numbers of crossing platform.

### 2.5 Transmission electron microscope

Mice on Day 7 after surgery were perfused with PBS followed by 4% paraformaldehyde and 0.25% glutaraldehyde after deep anaesthesia. Hippocampus tissues were removed and kept in 2.5% glutaraldehyde, and then, they were cut to 1 mm^3^ blocks with vibratome. The blocks were post‐fixed in 1% osmium tetroxide for 1 h, dehydrated in graded ethanol and embedded in epoxy resin. Polymerization was performed at 80°C for 24 h. The blocks were cut on an ultramicrotome into 60 nm slices, which were post‐stained with uranyl acetate and lead citrate staining, and viewed under a Hitachi 7100 electron microscopy.

### 2.6 *q*‐PCR

Total RNA of hippocampus tissues on Day 7 after surgery or cultured BV‐2 microglia was extracted using the TRIzol method. Then, the collected RNA was reverse‐transcribed to cDNA with the reverse transcription kit according to the manufacturer's instructions. The polymerase chain reaction quantification was carried out using the SYBR Green kit by the QuantStudio 5 (Applied Biosystems). The cycle threshold (CT) values of target genes were collected and normalized to GAPDH, and the fold changes of gene expression were calculated with the 2^−ΔΔCT^ method. The primer sequences used were showed in Table 1.

**TABLE 1:** Primer sequences.

Gene	Forward sequence	Reverse sequence
*miR‐124‐3p*	5′‐TCTTTAAGGCACGCGGTG‐3′	5′‐TATGGTTTTGACGACTGTGTGAT‐3′
*bdnf*	5′‐CCCGGTGTCGCCCTTAAAAA‐3′	5′‐CTCACCTGGTGGAACTTCTTTG‐3′
*ng*	5′‐AGCATCGTACAAACCCACCC‐3′	5′‐AAAACGTTCAGCTCTGGCCTA‐3′
*vilip‐1*	5′‐TGCTGGAGATTATCGAGGCTA‐3′	5′‐GGAAGGGTCGCTTTTTGCAG‐3′
*ym*	5′‐AGAAGGGAGTTTCAAACCTGGT‐3′	5′‐CTCTTGCTGATGTGTGTAAGTGA‐3′
*arg‐1*	5′‐CTCCAAGCCAAAGTCCTTAGAG‐3′	5′‐AGGAGCTGTCATTAGGGACATC‐3′
*il‐10*	5′‐CCGAGATGCCTTCAGCAGAGT‐3′	5′‐GGAGTTCACATGCGCCTTGAT‐3′
*tnf‐α*	5′‐GTAGCCCACGTCGTAGCAAA‐3′	5′‐ACAAGGTACAACCCATCGGC‐3′
*il‐1β*	5′‐AGAGCCCATCCTCTGTGACT‐3′	5′‐GCTCATATGGGTCCGACAGC‐3′
*il‐6*	5′‐AACGATGATGCACTTGCAGA‐3′	5′‐TCTCTCTGAAGGACTCTGGCT‐3′
*cyclin D1*	5′‐GGAGCAGAAGTGCGAAGA‐3′	5′‐GGGCCGGATAGAGTTGTC‐3′
*gapdh*	5′‐TGCCACTCAGAAGACTGTGG‐3′	5′‐GGATGCAGGGATGATGTTCT‐3′

### 2.7 Immunofluorescence

Mice on Day 7 after surgery were euthanized and perfused with sterile saline and 4% paraformaldehyde. Hippocampus of mice was quickly removed and post‐fixed in 4% paraformaldehyde for 1 day, and then moved into 30% sucrose solution for dehydration. Next, hippocampus transverse frozen sections (20 μm thick) were cut using freezing microtome (Leica, Germany) and were incubated with 5% goat serum for 2 h at room temperature. Hippocampus sections were then incubated with primary antibodies overnight at 4°C. Before incubation with secondary antibodies, the sections were washed using PBS for 3 times. After incubation with secondary antibodies for 2 h, digital images were captured with a high‐resolution fluorescence microscope equipped with a high‐resolution CCD Spot camera and then merged by Adobe Photoshop software. The antibodies used were listed as follows: rabbit cyclin D1 (1:200, Abcam, ab16663), mouse IBA‐1 (1:100, Abcam, ab283319), goat anti‐rabbit IgG (1:500, Abcam, ab150083) and goat anti‐mouse IgG (1:500, Abcam, ab150113).

### 2.8 Data acquisition and analysis

The bioinformatics data were acquired from the Gene Expression Omnibus (GEO) database. Hippocampus, microglial exosomes and non‐coding RNA profiling were used as key words to search the related miRNAs datasets. Finally, we chose two eligible microarray datasets GSE95070 and GSE133997 for further analysis. GSE95070 dataset contains five paired hippocampus tissues from postoperative cognitive dysfunction (POCD) and control mice. In GSE133997 dataset, we used the microglial exosome miRNAs sequencing data from three paired hippocampus tissues in the chronic phase of 42 days post traumatic brain injury mice (42DPI) and control. The differently expressed miRNAs were identified by limma package in R with *p* < 0.05. The |log_2_‐fold change (FC)| cut‐offs were set at 0.3 and 1 for GSE95070 and GSE133997, respectively. The differentially expressed miRNAs are visualized in volcano plots. The online Venn diagram tool was used to acquire the common differently expressed miRNAs between the two datasets (http://www.ehbio.com/test/venn/).

### 2.9 Isolation of microglial exosomes in hippocampus

Mice were euthanized and perfused with sterile PBS through the heart on Days 0, 3, 7 and 14 after surgery. Hippocampus tissues were obtained and incubated in Hibernate‐A cell culture medium (Thermo Fisher Scientific, USA); then, they were digested by 20 U/mL papain for 15 min at 37°C and blew to remove tissue fragment. Subsequently, samples were centrifuged at 2000 *g* for 10 min and 10,000 *g* for 30 min at 4°C to clear cell debris. Supernatants were obtained and filtered through a 0.22 mm filter gauze (Millipore Sigma, USA) to clear large particles. Then, total exosome isolation reagent was added to the supernatants at the proportion of 1:2 for incubating overnight at 4°C. Samples were collected the next day and ultracentrifuged at 100,000 *g* for 70 min at 4°C. Exosome precipitates were re‐suspended with calcium and magnesium free Dulbecco's PBS. For isolation of microglial exosomes, samples were incubated with 1.5 mg anti‐mouse CD11b antibody (Thermo Fisher Scientific) in 50 μL 3% BSA for 60 min. Then, the mixture was incubated with 10 μL Pierce Streptavidin Plus UltraLink Resin (Thermo Fisher Scientific) in 40 μL 3% BSA for 30 min. Then, the mixture was centrifuged at 800 *g* for 10 min at 4°C, and the microglial exosome precipitates were collected for following experiments.

### 2.10 BV‐2 microglia cell line culture and miR‐124‐3p mimic transfection

The BV‐2 microglia cell line was obtained from Naval Medical University and seeded in a 12‐well plates. The complete medium consisted of 95% DMEM/F12 medium, 10% foetal bovine serum, 100 U/mL penicillin and 100 mg/mL streptomycin. Microglia were cultured in an incubator in the condition of 5% CO_2_ at 37°C. When the density of BV‐2 microglia reached 10^5^/cm,^2^ miR‐124‐3p mimics (5′‐UAAGGCACGCGGUGAAUGCCCA‐3′, OBiO, Shanghai) were transfected into BV‐2 microglia to explore the effect of exosome miR‐124‐3p on microglia polarization. Firstly, miR‐124‐3p mimics were diluted and incubated with Lipofectamine 2000 (Invitrogen, USA) in serum‐free DMEM/F12 medium for 20 min; then, the transfection mixture was added into the plate for transfection for 6 h and replaced by complete medium for another 36 h culture. Subsequently, the cultured BV‐2 microglia were digested and centrifuged to separate BV‐2 microglia and culture medium. The BV‐2 microglia and culture medium samples were centrifuged at 2000 *g* for 10 min and 10,000 *g* for 30 min at 4°C to clear cell debris. Supernatants were obtained and filtered through a 0.22 mm filter gauze (Millipore Sigma, USA) to clear large particles. Then, total exosome isolation reagent was added to the supernatants at the proportion of 1:2 for incubating overnight at 4°C. Samples were collected the next day and ultracentrifuged at 100,000 *g* for 70 min at 4°C to acquire the exosome precipitates. miR‐124‐3p levels in exosomes obtained from BV‐2 microglia and culture medium were both identified by *q*‐PCR to observe the miR‐124‐3p changes in microglia and microglial exosomes, separately. The collected microglial exosomes overexpressing miR‐124‐3p were stored at 4°C and further injected stereotactically in hippocampus at 11:00 in the morning on Day 0 after surgery for experiments in vivo. Then, Morris water maze tests and tissue collection were carried out on Day 7 after surgery.

### 2.11 Statistical analysis

Statistical analyses were carried out using the IBM SPSS Statistics 23.0, and data were presented as mean ± SEM. Two‐way repeated‐measure ANOVA was used to compare escape latency, duration in target quadrant and numbers of crossing platform among different groups at different time points. The differences in two groups were compared by paired *t*‐test while the differences among three groups were compared by one‐way ANOVA analysis of variance followed by Dunnett's *t*‐test. A value of *p* < 0.05 was considered to be statistically significant.

## 3 RESULTS

### 3.1 Postoperative pain facilitated cognitive impairment in elderly mice

**FIGURE 1:**
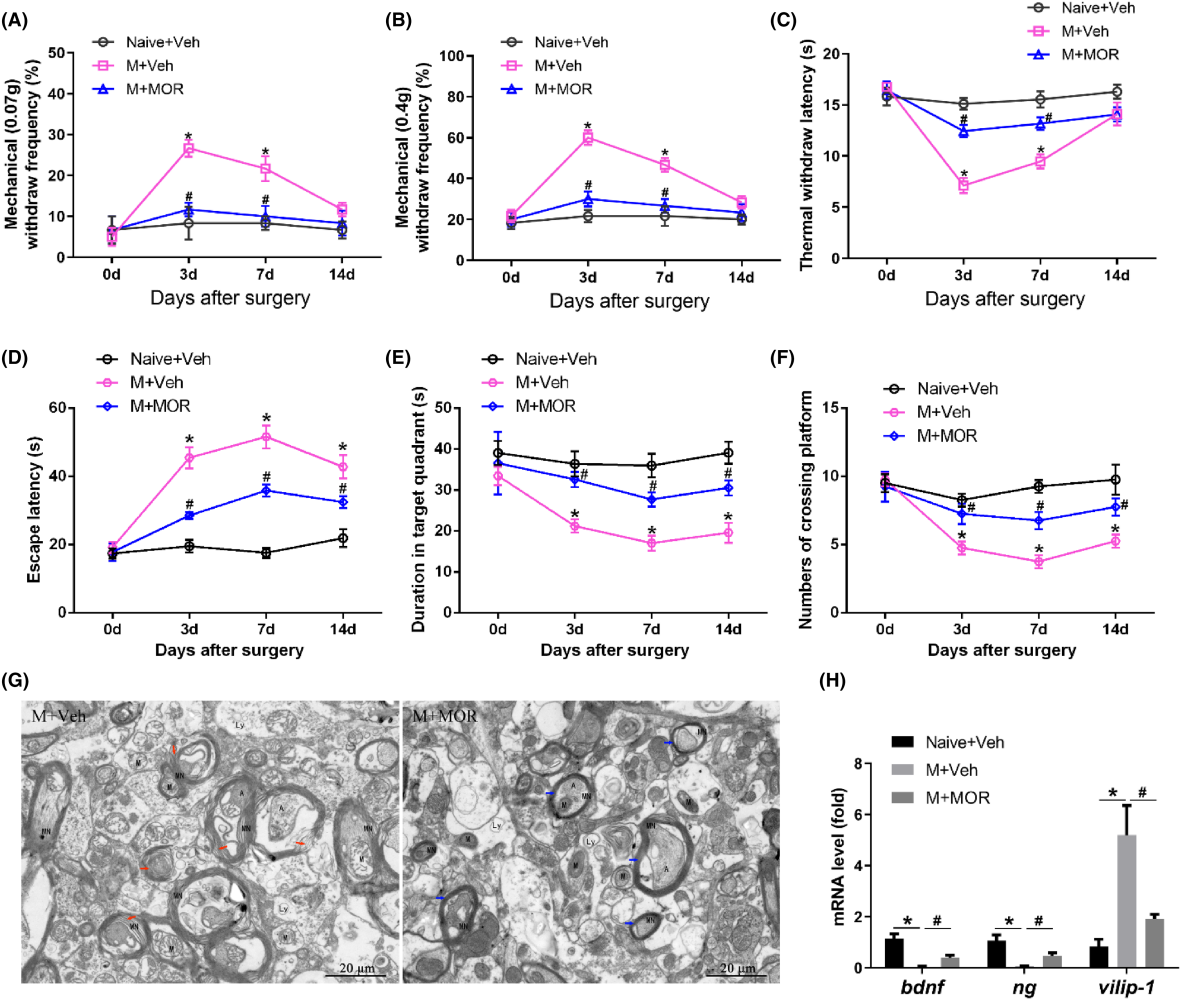
Postoperative pain facilitated cognitive impairment in elderly mice. (A) Mechanical withdraw frequencies to 0.07 g; (B) mechanical withdraw frequencies to 0.4 g; (C) thermal pain thresholds; (D) escape latency; (E) duration in target quadrant; (F) numbers of crossing platform; (G) transmission electron microscope of hippocampus, red arrows represent loose and demyelinated axonal myelin, blue arrows represent dense and normal axonal myelin; (H) mRNA levels of bdnf, ng and vilip‐1. Bar represents mean ± SEM, *n* = 6. *p < 0.05 compared with Naive+Veh group on the same day, ^#^ *p* < 0.05 compared with M + Veh group on the same day.

To evaluate the successful establishment of postoperative pain model in elderly mice, we firstly measured the mechanical and thermal pain thresholds in three groups. Mechanical and thermal pain thresholds were significantly decreased on Days 3 and 7 in M + Veh mice compared with that in Naive + Veh mice, while MOR analgesia significantly increased mechanical and thermal pain thresholds on Days 3 and 7 (Figure 1A–C), suggesting the successful establishment of postoperative pain model. To investigate the effects of postoperative pain on cognitive impairment in elderly mice, we used Morris Water Maze to evaluate the learning ability and spatial memory function. The escape latency was significantly increased while the time spent in the target quadrant and the number of platform crossings significantly decreased on Days 3, 7 and 14 in M + Veh mice compared with that in Naive + Veh mice, and the most pronounced cognitive impairment was observed on Day 7. MOR analgesia significantly decreased the escape latency and increased the duration in target quadrant and numbers of crossing platform compared with that in M + Veh mice (Figure 1D–F), suggesting that postoperative pain actually damaged the cognitive function in elderly mice. In the following experiments, we chose mice 7 days after surgery as the stable cognitive impairment model induced by postoperative pain. It was well known that cognitive function was mainly regulated by hippocampus, and we then examined the changes in neuronal structures in hippocampus by transmission electron microscope. In M + Veh mice, the axonal myelin lamina presented loose and demyelinated changes, while M + MOR mice presented dense and normal axonal myelin (Figure 1G). Moreover, the mRNA levels of neurotrophic factors (BDNF, NG) were significantly down‐regulated while the neurodegeneration marker VILIP‐1 was up‐regulated in M + Veh mice compared with that in Naive + Veh mice, but MOR analgesia up‐regulated BDNF and NG and down‐regulated VILIP‐1 in hippocampus (Figure 1H), suggesting that postoperative pain facilitated neural damages in hippocampus of elderly mice.

### 3.2 Postoperative pain promoted the M1 polarization of microglia in hippocampus

**FIGURE 2:**
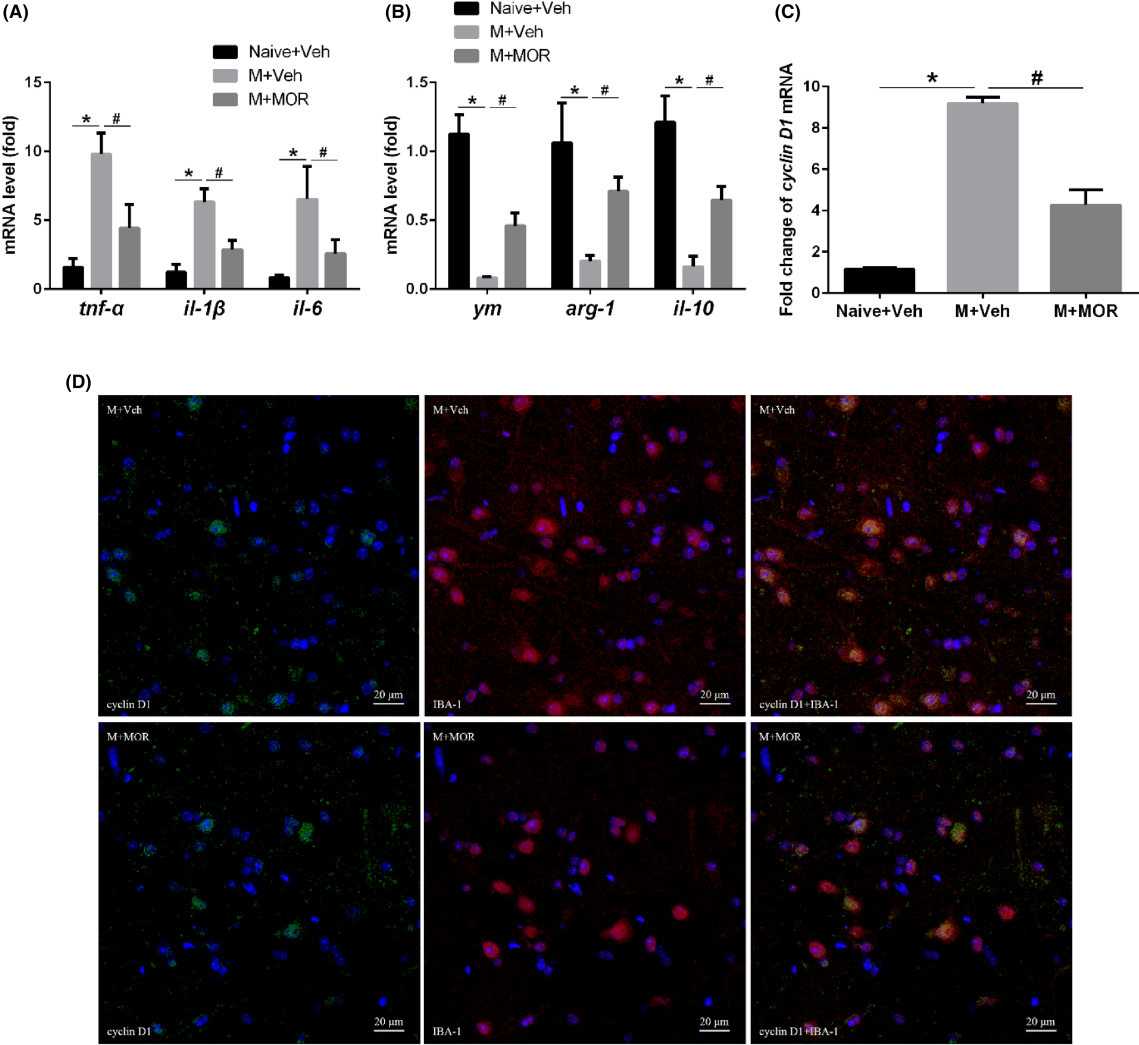
Postoperative pain promoted the M1 polarization of microglia in hippocampus. (A) mRNA levels of pro‐inflammatory markers (tnf‐α, il‐1β, il‐6); (B) mRNA levels of anti‐inflammatory markers (ym, arg‐1, il‐10); (C) mRNA level of cyclin D1; (D) double staining of cyclin D1 and IBA‐1. Bar represents mean ± SEM, *n* = 6. *p < 0.05 compared with Naive+Veh group, ^#^ *p* < 0.05 compared with M + Veh group.

Microglia are widely present in hippocampus and mainly involve in neuronal remodelling and immune response, and M1 polarization of microglia actively promotes neuroinflammation. We next explored whether postoperative pain affect the polarization of microglia in hippocampus. *q*‐PCR results showed that there was a significant increase in the levels of pro‐inflammatory factors TNF‐α, IL‐1β and IL‐6 while the anti‐inflammatory factors Ym, Arg‐1 and IL‐10 decreased in M + Veh mice compared with that in Naive+Veh mice, but MOR analgesia significantly reversed the changes (Figure 2A,B). Microglia proliferation marker cyclin D1 also increased significantly in M + Veh mice compared with that in Naive + Veh mice, while it significantly decreased in M + MOR mice compared with that in M + Veh mice (Figure 2C). In M + MOR mice compared to M + Veh mice, there was a noticeable decrease in the co‐expression of cyclin D1 and the microglia activation marker IBA‐1 in the hippocampus (Figure 2D), suggesting a potential effect of postoperative pain on the M1 polarization and activation of microglia in hippocampus.

### 3.3 Postoperative pain decreased microglial exosome miR‐124‐3p level in hippocampus

**FIGURE 3:**
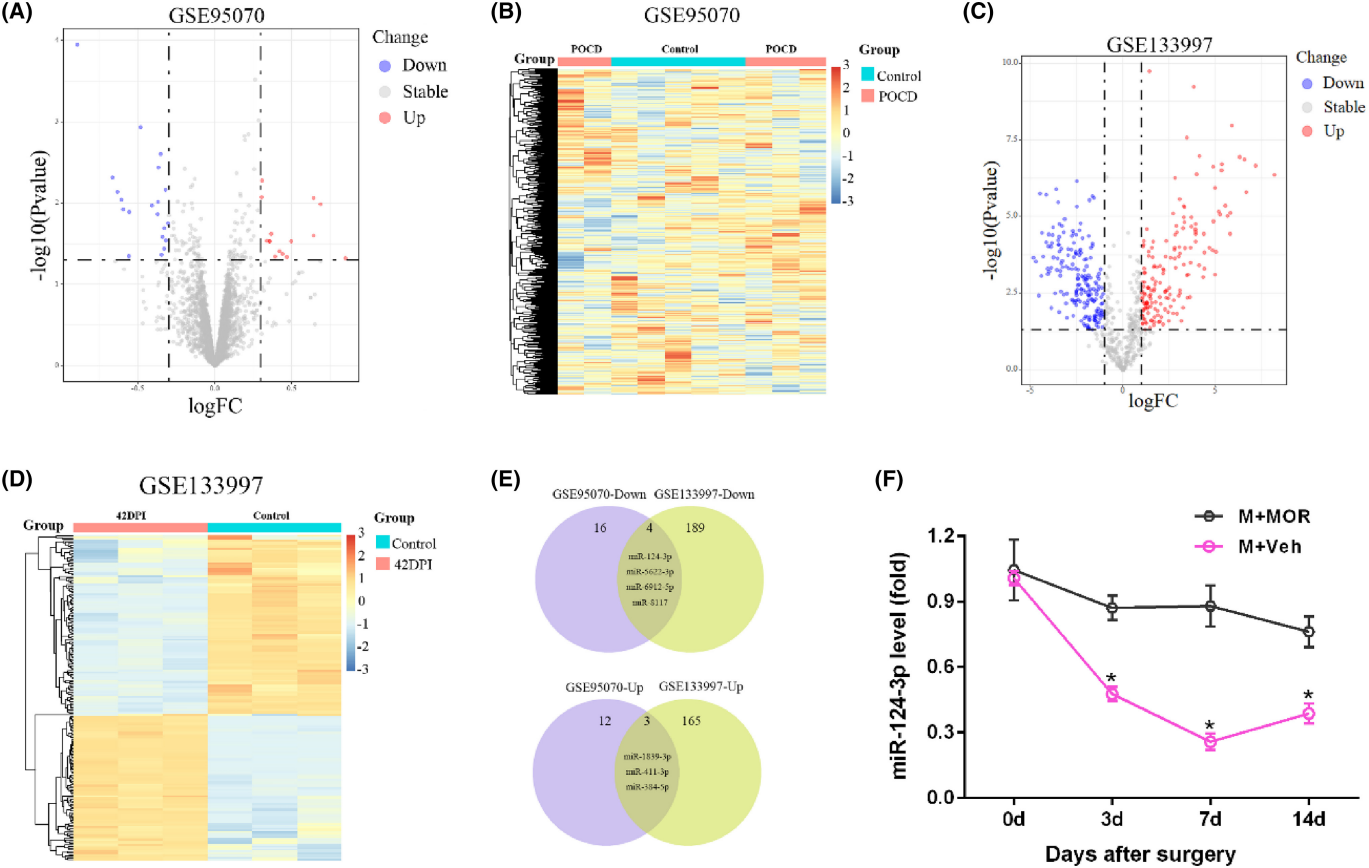
Postoperative pain decreased microglial exosome miR‐124‐3p level in hippocampus. (A) Volcano plot in GSE95070 datasets; (B) heatmap in GSE95070 datasets; (C) volcano plot in GSE133997 datasets; (D) heatmap in GSE133997 datasets; (E) venn diagram in differently expressed miRNAs of GSE95070 and GSE133997; (F) miR‐124‐3p levels in hippocampus after surgery. Bar represents mean ± SEM, *n* = 6. *p < 0.05 compared with M + MOR group on the same day.

To study the roles of microglial exosome miRNAs in the pathological changes, we searched the GEO database to identify the miRNAs changes, and finally, we chose GSE95070 and GSE133997 for further analysis. After standardizing the microarray results, 28 differently expressed miRNAs in control and POCD mice were identified in GSE95070 with 16 down‐regulated and 12 up‐regulated miRNAs (Figure 3A,B), while 354 differently expressed miRNAs in control and 42DPI mice were screened out in GSE133997 with 189 down‐regulated and 165 up‐regulated miRNAs (Figure 3C,D). After taking the intersection of Venn diagram in differently expressed miRNAs of GSE95070 and GSE133997, a total of seven common differently expressed miRNAs were identified, including four common down‐regulated miRNAs (miR‐124‐3p, miR‐5622‐3p, miR‐6912‐5p and miR‐8117) and three common up‐regulated (miR‐1839‐3p, miR‐411‐3p and miR‐384‐5p) (Figure 3E). As miR‐124‐3p has been reported to possess neuroprotective effects,^34^, ^35^ we next explored the microglial exosome miR‐124‐3p level in hippocampus of mice experiencing postoperative pain. As shown in Figure 3F, the microglial exosome miR‐124‐3p level in the hippocampus was significantly decreased on Days 3, 7 and 14 after surgery in M + Veh mice compared with that in M + MOR mice.

### 3.4 miR‐124‐3p overexpression alleviated M1 polarization of microglia in vitro

**FIGURE 4:**
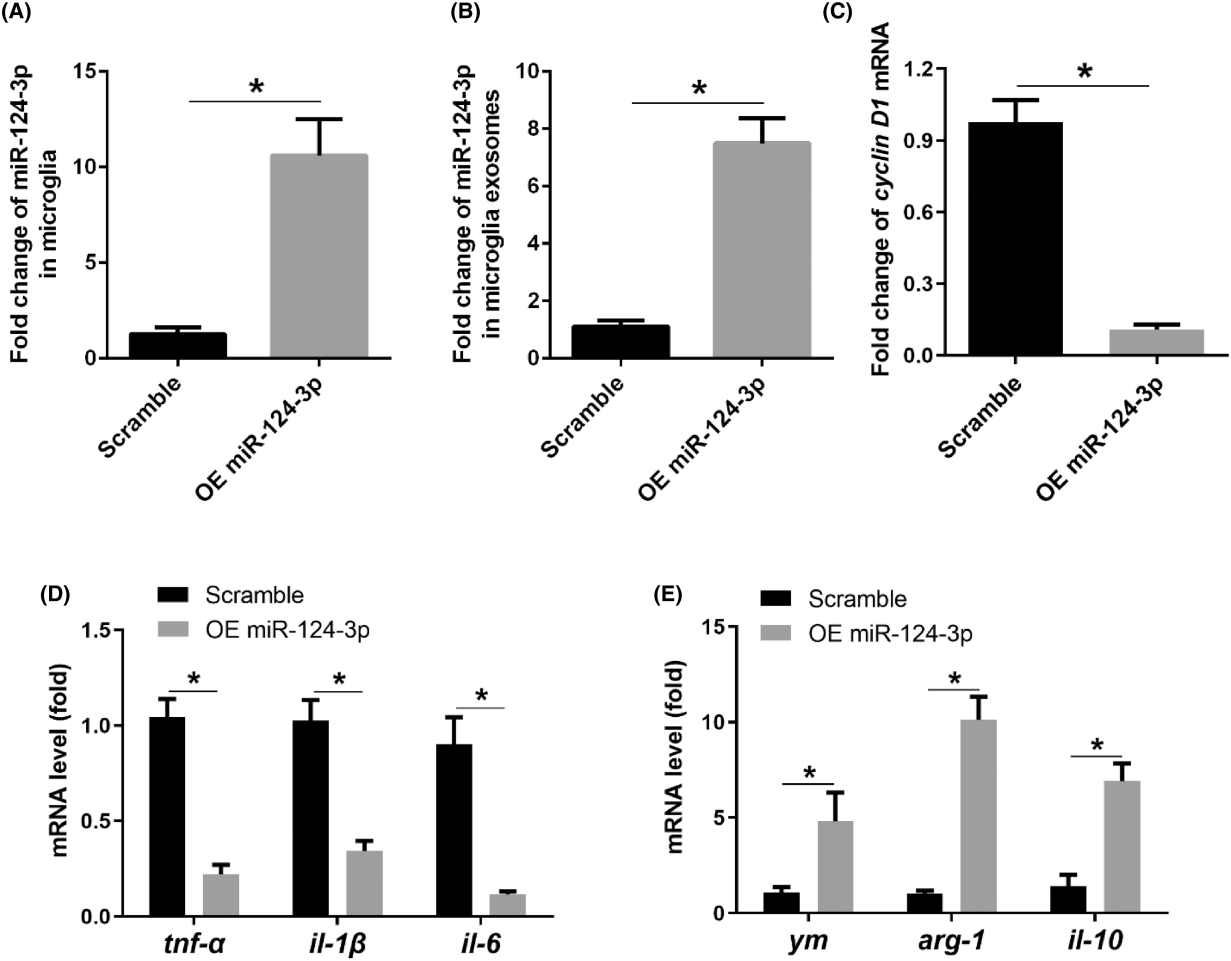
miR‐124‐3p overexpression alleviated M1 polarization of microglia in vitro. (A) miR‐124‐3p level in BV‐2 microglia after miR‐124‐3p mimic transfection; (B) miR‐124‐3p level in BV‐2 microglial exosomes after miR‐124‐3p mimic transfection; (C) mRNA level of cyclin D1; (D) mRNA levels of pro‐inflammatory markers (tnf‐α, il‐1β, il‐6); (E) mRNA levels of anti‐inflammatory markers (ym, arg‐1, il‐10). Bar represents mean ± SEM, *n* = 6. *p < 0.05 compared with Scramble group.

To further investigate the effects of exosome miR‐124‐3p on microglia polarization, we conducted overexpression of miR‐124‐3p in cultured BV‐2 microglia cell line and detected microglia polarization. miR‐124‐3p overexpression in microglia was successfully conducted by increased miR‐124‐3p levels in BV‐2 microglia and microglial exosome precipitates (Figure 4A,B). Additionally, cyclin D1 and pro‐inflammatory factors TNF‐α, IL‐1β and IL‐6 significantly decreased, while the anti‐inflammatory factors Ym, Arg‐1 and IL‐10 increased in BV‐2 microglia overexpressing miR‐124‐3p compared with that in Scramble BV‐2 microglia (Figure 4C–E), suggesting that miR‐124‐3p overexpression alleviated M1 polarization and proliferation of microglia in vitro.

### 3.5 Stereotactic injection of exogenous microglial miR‐124‐3p in hippocampus alleviated M1 polarization of microglia in vivo

**FIGURE 5:**
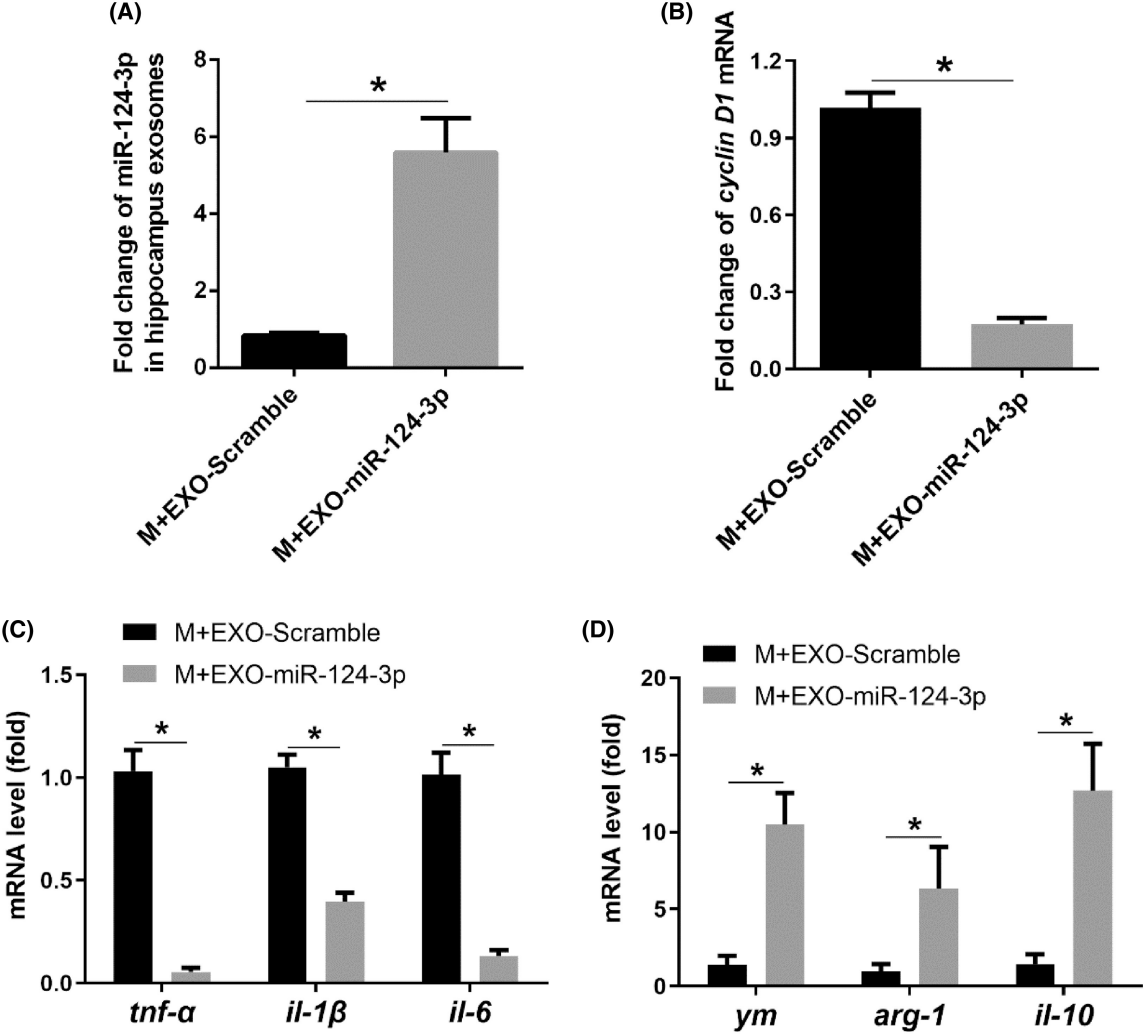
Stereotactic injection of exogenous microglial miR‐124‐3p in hippocampus alleviated M1 polarization of microglia in vivo. (A) miR‐124‐3p levels in hippocampus after stereotactic injection of exogenous microglial miR‐124‐3p; (B) mRNA level of cyclin D1; (C) mRNA levels of pro‐inflammatory markers (tnf‐α, il‐1β, il‐6); (D) mRNA levels of anti‐inflammatory markers (ym, arg‐1, il‐10). Bar represents mean ± SEM, *n* = 6. *p < 0.05 compared with M + EXO‐Scramble group.

Subsequently, we explored the function of exogenous microglial miR‐124‐3p on neuroinflammation in hippocampus by stereotactic injection. The collected exosome precipitates from BV‐2 microglia overexpressing miR‐124‐3p and Scramble BV‐2 microglia were both administrated into hippocampus, and the successful stereotactic injection was confirmed by increased miR‐124‐3p levels in hippocampus exosomes (Figure 5A). Also, cyclin D1 and pro‐inflammatory factors TNF‐α, IL‐1β and IL‐6 significantly decreased, while the levels of anti‐inflammatory factors Ym, Arg‐1 and IL‐10 increased after exogenous microglial miR‐124‐3p administration compared with Scramble administration in postoperative pain mice (Figure 5B–D), further confirming stereotactic injection of exogenous microglial miR‐124‐3p in hippocampus alleviated M1 polarization and proliferation of microglia in vivo.

### 3.6 Exogenous microglial miR‐124‐3p improved cognitive impairment induced by postoperative pain in elderly mice

**FIGURE 6:**
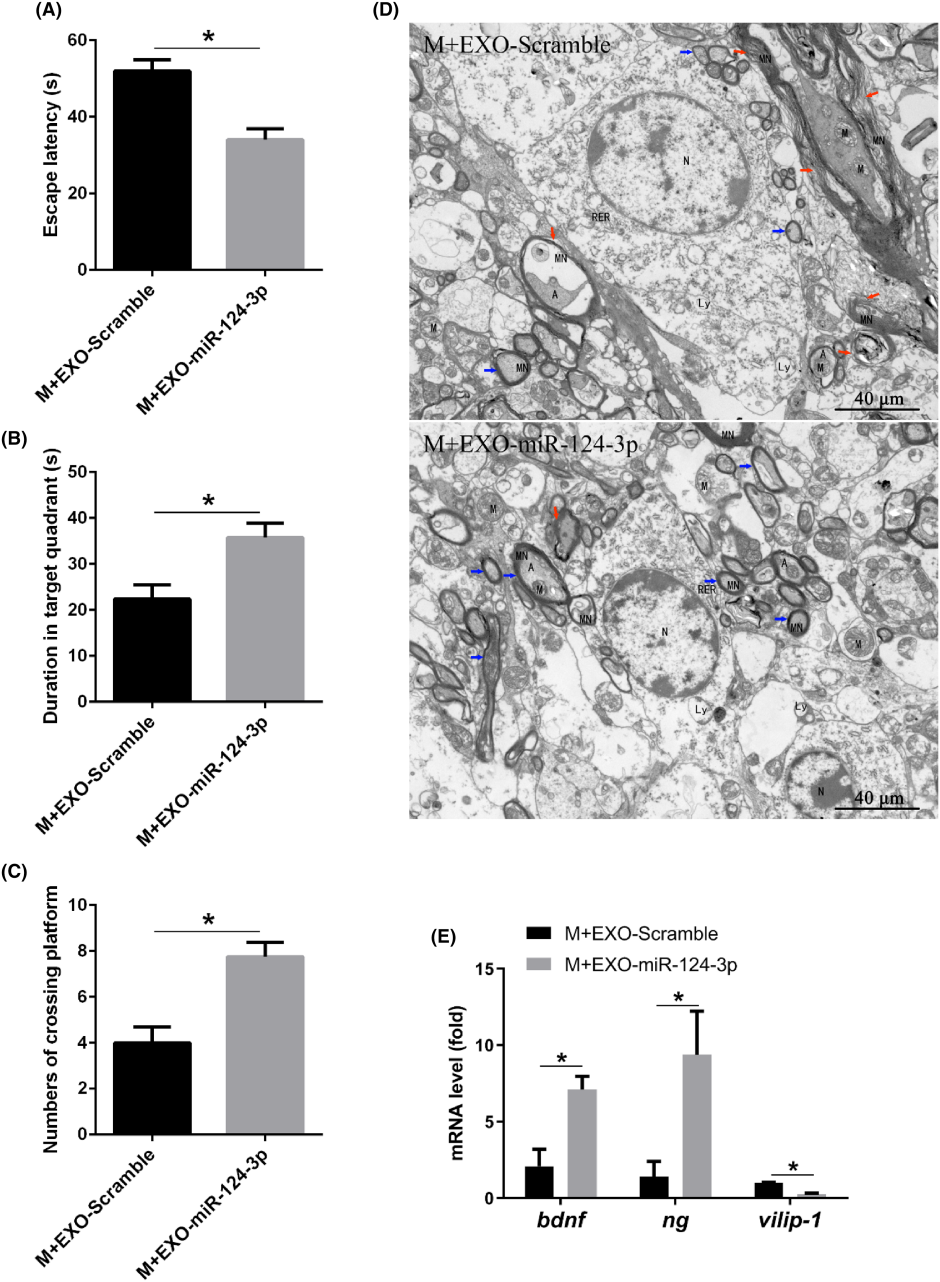
Exogenous microglial miR‐124‐3p improved cognitive impairment induced by postoperative pain in elderly mice. (A) Escape latency; (B) duration in target quadrant; (C) numbers of crossing platform; (D) transmission electron microscope of hippocampus, red arrows represent loose and demyelinated axonal myelin, blue arrows represent dense and normal axonal myelin; (E) mRNA level of bdnf, ng and vilip‐1. Bar represents mean ± SEM, *n* = 6. *p < 0.05 compared with M + EXO‐Scramble group.

Finally, we observed the function of exogenous microglial miR‐124‐3p on cognitive impairment induced by postoperative pain in elderly mice. By performing Morris Water Maze tests, we found that the escape latency was significantly decreased while the duration in target quadrant and numbers of crossing platform significantly increased after exogenous microglial miR‐124‐3p administration compared with Scramble administration in postoperative pain mice (Figure 6A–C), suggesting that exogenous microglial miR‐124‐3p improved learning ability and spatial memory function in postoperative pain mice. Transmission electron microscope showed that the axonal myelin lamina became dense and normal after exogenous microglial miR‐124‐3p administration (Figure 6D). Further, the mRNA levels of BDNF and NG were up‐regulated while VILIP‐1 was down‐regulated after exogenous microglial miR‐124‐3p administration compared with Scramble administration in postoperative pain mice (Figure 6E), suggesting that exogenous microglial miR‐124‐3p increased neuroprotective factors, which could be relevant to cognitive impairment induced by postoperative pain in elderly mice.

## 4 DISCUSSION

Cognitive impairment induced by postoperative pain in elderly patients gradually becomes an urgent problem that deteriorates the recovery during the perioperative period 15–18. Patients with cognitive impairment often present severe impairments in social activity, learning and memory, which is detrimental to the rehabilitation quality of elderly patients.^36^ Studies have confirmed that microglia polarization is of huge influence on neuronal activities and synaptic plasticity in the hippocampus, and finally affects the cognitive function.^37^, ^38^ We used bioinformatics analysis and identified the critical role of microglial exosome miR‐124‐3p in cognitive impairment. Fewer studies were focus on the protective mechanisms of miR‐124‐3p‐containing exosomes in the hippocampus on cognitive impairment. Here, we confirmed that postoperative pain actually induced cognitive impairment and axon demyelination, accompanied by microglia M1 polarization and decreased neuroprotective factors. Bioinformatics analysis and decreased microglial exosome miR‐124‐3p in postoperative pain mice both indicated the crucial role of exosome miR‐124‐3p in cognitive impairment, and overexpressing miR‐124‐3p in microglia reversed M1 polarization in vitro. Finally, stereotactic injection of exogenous microglial miR‐124‐3p in hippocampus alleviated M1 polarization of microglia, improved cognitive impairment and axon demyelination in vivo. These results all implied the protective effects of microglial exosome miR‐124‐3p on cognitive impairment through regulating microglia polarization in hippocampus of elderly mice.

Neuroinflammation is a common feature of most nervous dysfunctions like cognitive disorders, multiple sclerosis, Alzheimer's disease or pathological pain.^39^ Various types of cells are involved in the development of neuroinflammation in CNS, especially microglia and astrocyte.^40^, ^41^ Functioning as immune cells in CNS, activated microglia can activate inflammasome, NF‐κB and other inflammatory signalling pathways.^42^, ^43^ In the resting state, microglia sense the changes of microenvironments around synapses. In response to harmful stimuli, microglia activate a series of stress responses to shift into the activated state and M1 polarization.^44^ Abnormal microglial activation can contribute to the acceleration of neuronal autophagy and apoptosis during neurodegeneration.^45^ The present study also confirmed the close relationships between M1 polarization of microglia and cognitive impairment. In the process of M1 polarization, multiple signalling molecules were synthesized and released into the microenvironments around neurons, including exosomes which were rich in proteins, lipids, RNAs, etc. Aβ clearance plays a significant role in neurodegenerative diseases. Some studies indicate microglial exosomes having both beneficial and detrimental effects for the development of Aβ abnormalities and neurodegeneration in neurodegenerative diseases.^46^ In Alzheimer's disease, microglia activation acquired beneficial effects in the early stage by inducing phagocytosis and Aβ clearance with the help of microglial exosomes, while some substances in microglial exosomes damaged neurons in the latter stage, such as IL‐1β, soluble toxic Aβ peptides, caspase‐1 and others.^47^, ^48^ The bioinformatics analysis in this study identified the critical role of exosome miR‐124‐3p in cognitive impairment, and we also observed the decreased miR‐124‐3p level in hippocampus after surgery, which provided a potential target for investigating the mechanism of cognitive impairment induced by postoperative pain.

Being a highly specific miRNA in the CNS, miR‐124‐3p is a critical regulator modulator of immunity and inflammation, and is widely involved in microglia regulation, inflammatory responses, neuronal development and regeneration.^49^, ^50^ Prior studies have confirmed that microglia in hippocampus kept synthesizing, assembling and secreting various types of exosomes into synapses to regulate synaptic plasticity and neural activities.^51^, ^52^, ^53^ These exosomes in turn affects the microglia polarization, which was concordant with our results. In this study, we generated BV‐2 microglia that overexpressed miR‐124‐3p and confirmed the promotion of M2 polarization and reduced proliferation of microglia in vitro. Similarity, stereotactic injection of exogenous microglial miR‐124‐3p in hippocampus also alleviated M1 polarization and proliferation of microglia in vivo. We also confirmed that exogenous microglial miR‐124‐3p in hippocampus improved cognitive impairment, axon demyelination and neurotrophic factors. The potential mechanisms may be studied further in our continuous study, and the present results actually verified the conclusion that exosome miR‐124‐3p in microglia could exert protective effects in cognitive impairment induced by postoperative pain.

Some studies showed that miR‐124‐3p could directly regulate Rela gene to promote Aβ hydrolysis and inhibit Aβ abnormalities.^54^ As the phagocytes and immune cells in hippocampus, microglia are responsible for clearing Aβ and stabilizing the microenvironments. Xintong Ge found that the damaged neurons in hippocampus could absorb exogenous microglial exosome miR‐124‐3p by intravenous injection in the traumatic brain injury mouse model.^34^, ^55^ Exosome miR‐124‐3p in hippocampal microenvironments can also alter the expression of various neurotrophic factors, which are essential to neural growth. For example, BDNF can promote the growth and differentiation of neurons and enhance synaptic plasticity, and dysfunction in BDNF synthesis is an important risk factor for Alzheimer's disease or age‐related cognitive impairment.^56^ NG, a calcium‐binding protein mainly expressed in dendritic spines, plays a neuroprotective role in the pathological process of cerebral ischemia.^57^ VILIP‐1, a neuron‐specific calcium‐sensitive protein, promotes autophagy and apoptosis of neurons by regulating the activity of adenylate cyclase, which can be used as a biomarker of neurodegeneration.^58^ Microglial exosome miR‐124‐3p may also be absorbed by nearby neurons and regulate the transcription and translation of neurotrophic factors to affect cognitive impairment, which may be further studies by our following experiments.^59^

In conclusion, microglial exosome miR‐124‐3p in hippocampus is beneficial to alleviating cognitive impairment induced by postoperative pain through regulating microglia polarization in elderly mice. Consequently, exogenous exosome miR‐124‐3p may be potential therapeutic strategies for cognitive impairment induced by postoperative pain in elderly patients.

## AUTHOR CONTRIBUTIONS

**Erliang Kong:** Writing – original draft (equal). **Xuqiang Geng:** Writing – original draft (equal). **Feixiang Wu:** Data curation (equal). **Wei Yue:** Data curation (equal); formal analysis (equal); resources (equal). **Yuming Sun:** Conceptualization (equal). **Xudong Feng:** Conceptualization (equal).

## FUNDING INFORMATION

This research was funded by Henan provincial Natural Science Foundation of China (222300420384), Natural Science Foundation of Shanghai of China (19ZR1400400), Henan provincial Medical Science and Technology Research Project (LHGJ20220920), (SBGJ202003056) and (SBGJ202102204) and Youth Scientific Research Project of Anesthesia Quality Control Center of Henan Province.

## CONFLICT OF INTEREST STATEMENT

The authors declare that there were no competing interests.

## Data Availability

The datasets used and analysed during the current study are available from the corresponding author on reasonable request.
